# Onchocerciasis in the Americas: from arrival to (near) elimination

**DOI:** 10.1186/1756-3305-4-205

**Published:** 2011-10-25

**Authors:** Ken Gustavsen, Adrian Hopkins, Mauricio Sauerbrey

**Affiliations:** 1 Corporate Responsibility, Merck. 1 Merck Drive, WS2A-56, Whitehouse Station, New Jersey, 08889, USA; 2 MECTIZAN Donation Program. 325 Swanton Way, Decatur, Georgia, 30030, USA; 3 Onchocerciasis Elimination Program for the Americas. 14 Calle 3-51, Zona 10, Edificio Murano Center, Oficina 1401, Guatemala City, 01010, Guatemala

**Keywords:** onchocerciasis, river blindness, neglected tropical diseases, Latin America, parasites, ivermectin, disease elimination

## Abstract

Onchocerciasis (river blindness) is a blinding parasitic disease that threatens the health of approximately 120 million people worldwide. While 99% of the population at-risk for infection from onchocerciasis live in Africa, some 500,000 people in the Americas are also threatened by infection. A relatively recent arrival to the western hemisphere, onchocerciasis was brought to the New World through the slave trade and spread through migration. The centuries since its arrival have seen advances in diagnosing, mapping and treating the disease. Once endemic to six countries in the Americas (Brazil, Colombia, Ecuador, Guatemala, Mexico and Venezuela), onchocerciasis is on track for interruption of transmission in the Americas by 2012, in line with Pan American Health Organization resolution CD48.R12. The success of this public health program is due to a robust public-private partnership involving national governments, local communities, donor organizations, intergovernmental bodies, academic institutions, non-profit organizations and the pharmaceutical industry. The lessons learned through the efforts in the Americas are in turn informing the program to control and eliminate onchocerciasis in Africa. However, continued support and investment are needed for program implementation and post-treatment surveillance to protect the gains to-date and ensure complete elimination is achieved and treatment can be safely stopped within all 13 regional foci.

## Introduction

Onchocerciasis is a tropical disease caused by the parasite *Onchocerca volvulus*, which is transmitted by the black fly. The *Simulium *flies that transmit onchocerciasis breed in fast-flowing rivers, giving rise to the common name "river blindness" for the disease. Once transmitted to a human host, L_3 _infective-stage larvae molt twice and mature into adult worms within nodules underneath the skin, commonly in the midsection and around the head. The adult worms inside reproduce, creating millions of larvae (microfilariae) that move under the skin and cause intense itching, skin lesions and loss of pigmentation, as well as penetrating the eye causing sight impairment and eventual blindness.

Worldwide there are more than 120 million people at risk of contracting the disease, with some 18 million people infected. More than 99% of the disease burden is in Africa. In the Americas, about 500,000 people have historically been at risk of infection in 13 foci throughout Brazil, Colombia, Ecuador, Guatemala, Mexico and Venezuela.

## An Imported Infection

Onchocerciasis arrived in the Americas through the slave trade. Starting in the early 16^th ^century, slaves from the heavily endemic areas of West Africa were brought to Central and South America, bringing with them *O. volvulus *parasites. Slaves then migrated within the colonies, including between coffee plantations, carrying with them the parasites with which they were infected. Not being confined to the original African hosts, and as suitable *Simulium *species were present, the parasites were also transmitted to the indigenous American population and then spread further through migration, including among certain contiguous border countries like Guatemala and Mexico [1]. The spread of the disease through labor and other migration, and the presence of different vectors in the environment, explains the presence of onchocerciasis in Ecuador, Colombia, Guatemala, Mexico and Northern Venezuela [2], and genetic testing of parasites confirms this linkage between Old World and New World *O. volvulus *[3].

However, one open question is the manner in which onchocerciasis has spread to the Amazon region in the border area between Brazil and Venezuela. This area is extremely isolated, is not part of the coffee growing region and is not part of common trade routes, either now or historically. One theory is that the geographic area which the Amazonian people groups once inhabited was much more extensive in the past, encompassing areas where there was once sufficient contact for onchocerciasis transmission to occur from slave to native populations. In the years following that exposure, however, these people groups moved into more isolated regions that had no outside contact and thus brought onchocerciasis into this isolated, interior area. Another theory is that it came through movement of indigenous populations from other foci from the North of Venezuela [4].

Although the slave trade brought onchocerciasis to the Americas, *Simulium damnosum*, the most common vector that transmits *O. volvulus *in West Africa, is not found in the Americas. Instead, onchocerciasis in the Northern part of the Americas, Guatemala and Mexico, is primarily transmitted by *S. ochraceum*, which is considered less efficient than *S. damnosum *at transmitting *O. volvulus*. On the other hand, disease is transmitted in the southern part, namely Brazil, Colombia, Ecuador, and Southern Venezuela, by *S. exiguum *and *S. guianense*, which are both considered as efficient as *S. damnosum*. All of them, however, do not cover extensive geographical areas. As a result, onchocerciasis exists in the Americas only in circumscribed areas that are relatively small and isolated. From a public health standpoint, this means that onchocerciasis in the Americas has been easier to track, map and treat, and less likely to spread, than it is in Africa. In addition, the treatment strategy adopted by OEPA for the Americas involves mass drug administration to all the eligible population at least twice per year, versus once a year as it is done in Africa.

Given the impact of onchocerciasis in the Americas it is no surprise that there was critical early scientific investigation into the disease conducted by a researcher from an affected country. A physician from Guatemala, Dr. Rodolfo Robles (1878-1939), conducted studies on patients with onchocerciasis, which led to the discovery in 1915 that the disease is caused by *O. volvulus*. In honor of this important research contribution, onchocerciasis is also called 'Robles Disease' [5].

## Treatment Options

There have been various treatment approaches to control the disease in individual patients and at the community level in the Americas. Surgical removal of the subcutaneous nodules that contain the adult parasites was conducted mainly in the past. However, not all nodules are easily visible and palpable since some of them are deeper in the tissue, making removal difficult and frequently missed, and also removal, when possible, is painful to the patient. As a result, surgical control of onchocerciasis is difficult to conduct on a large-scale, community level. The first pharmaceutical treatments used were diethylcarbamazine (DEC) and suramin. However, treatment with these two drugs can create serious side effects, including blindness in patients with advanced onchocercal eye disease and renal failure in the case of suramin, making them unattractive treatment options, especially for wide-scale use at a community level [6]. Vector control, through the use of insecticides to control the black fly population, has also been used with some success on a local level [7]. The current MDA treatment approach, initiated in the late 1980s following the discovery and development of MECTIZAN (ivermectin) by the pharmaceutical company Merck (known outside the United States and Canada as MSD), has offered the opportunity for disease control and elimination.

## The River Blindness Partnership

In 1987 Merck announced its decision to donate MECTIZAN - "as much as necessary for as long as necessary" - through the MECTIZAN Donation Program. In support of this commitment a global partnership developed involving the MECTIZAN Expert Committee, local communities affected by onchocerciasis, local and international non-profit organizations, national governments and their ministries of health, the World Health Organization, and the World Bank [8].

In the Americas, the effort to eliminate onchocerciasis is carried out through a broad range of partners under the general coordination of the Onchocerciasis Elimination Program for the Americas (OEPA). Led and supported by the Carter Center, OEPA - headquartered in Guatemala - is notable for the ongoing, direct involvement of ministries of health from the six affected countries. The OEPA partnership involves financial and technical support through the Bill & Melinda Gates Foundation, the US Centers for Disease Control and Prevention, the Lions Clubs International Foundation, the Pan American Health Organization and others. The various members of the partnership engage on key issues such as overall organization and alignment of roles, advocacy, financial support and drug donations, support for operational and technical research, education, and communications [9].

## Operational Efficiencies

Through ongoing feedback between field programs and other members of the partnership, in Africa and the Americas, various operational and technical efficiencies have been incorporated over the past two decades. For example, the packaging of MECTIZAN was changed from an individually packed foil strip to a 500-count bottle, allowing for simpler distribution to communities and less waste. The MECTIZAN tablet size was reduced from 6 mg to 3 mg, enabling more accurate and simple dosing at the field level without the need to split the tablet for those requiring a 3 mg or 9 mg dose. The determination of the proper dose for each treatment is now determined by height rather than weight, making use of a low-tech measuring stick instead of a bulky and unwieldy scale [10]. Also in 2003, Merck authorized treatment in children by the use of either one of the three criteria normally required: weight, height and/or age, which provided the opportunity to increase the number of eligible children receiving treatment. Early in the program the treatment frequency strategy in the Americas was established to be twice annually after it was demonstrated that this would have an accelerating effect on stopping disease transmission [11]. These improvements, in addition to the fact that the MDA approach is offered at least twice each year to all eligible individuals living in all endemic communities with the goal of reaching a minimum of 85% treatment coverage, have all supported the advancement of the program objective in the Americas from one of simply controlling onchocerciasis, to one of completely eliminating transmission of the disease.

## Consensus for Action

**Figure 1:**
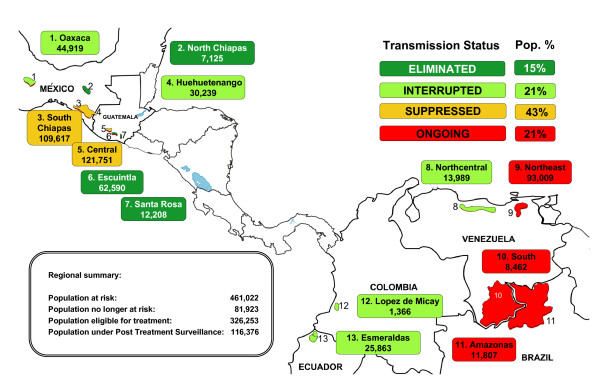
**Geographic distribution and transmission status of the 13 onchocerciasis foci in the Americas as of December, 2010**.

Further galvanizing the resolve of the partners to eliminate onchocerciasis from the Americas was a resolution by the Pan American Health Organization (PAHO) in 1991, calling for the elimination of onchocerciasis morbidity from the Americas by 2007 [12]. PAHO Resolution XIV served as an effective advocacy platform, providing the springboard from which OEPA was launched in 1992. The main goal of Resolution XIV was attained in most, but not all, of the 13 foci by 2007, providing much needed regional attention on the public health opportunity of onchocerciasis elimination. One country, Colombia (with its single focus, Lopez de Micay), did in fact achieve the goal of interrupting transmission of onchocerciasis in 2007, serving as a proof of concept (See Figure 1).

Recognizing the ongoing potential for complete elimination of onchocerciasis in the Americas region, PAHO Directing Council approved a new resolution in 2008 (CD48.R12). The goal of this resolution is three-fold: (1) to complete elimination of new onchocerciasis-related eye disease in all foci by 2012; (2) to interrupt onchocerciasis transmission in the 13 foci by 2012 (the last year for the distribution of treatment); (3) to complete the required three-year post-treatment epidemiological surveillance phase following WHO's criteria and procedures for certification of elimination of human Onchocerciasis by 2015 [13]. Suppression of transmission occurs when the population is no longer exposed to L_3 _infective-stage larvae through vector transmission, but treatment continues. Areas characterized as having interrupted transmission have achieved negative PCR (Polymerase Chain Reaction) in flies and negative OV16 antibody tests in young children, and where treatment has been suspended. Following a 3-year surveillance period, an ongoing negative PCR result indicates elimination of transmission [14].

**Figure 2:**
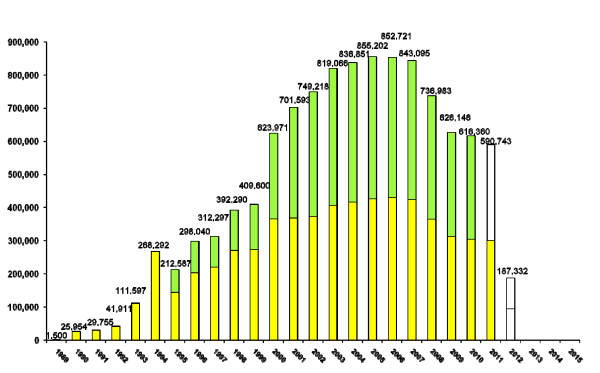
**History of MECTIZAN treatment in the Americas and projection from 2011 - 2015**. In 1995 all foci initiated 2x/year treatment. The annual peak of aggregate treatments for the region was reached in 2005. If the goals of PAHO resolution CD48.R12 are achieved, 2012 will be the final year that onchocerciasis treatment will be necessary, with all countries eligible for WHO certification of elimination no later than 2016.

Already, a peak has been reached in the number of treatments of MECTIZAN distributed in the Americas as individual foci reach endpoints of suspected interruption of transmission of onchocerciasis. Total treatments on a regional level reached a peak in 2003-2006 and are forecast to stop entirely in 2013 (see Figure 2). At the end of 2010, both Colombia and Ecuador had interrupted transmission and completely stopped ivermectin treatment on a national level and are undergoing the required post-treatment surveillance to confirm disease elimination in order to request certification by the World Health Organization. Certification for Colombia is expected at the end of 2011 or early 2012; Ecuador will be eligible by 2013. At a sub-national level, several formerly endemic areas in Mexico, Guatemala and Venezuela have also stopped treatment but will have to wait until all foci within each given country become ready, since according to WHO's criteria, only entire countries can request final certification. However, the Yanomami area on the Brazil-Venezuela border is considered the greatest challenge to meeting the ambitious goal set by Resolution CD48.R12 [14].

Continued attention to operational elements of the river blindness program in the Americas is highly necessary in order to achieve and maintain the elimination of transmission, which would allow for permanent suspension of treatment with MECTIZAN.

The most basic objective is for the remaining areas undergoing treatment with MECTIZAN to maintain high coverage - greater than 85% of the total population at-risk - for their bi-annual treatments, with treatment increased to 4 times per year in some areas where necessary and feasible to accelerate the transmission interruption process [7,14]. While the 85% threshold has now been reached in all foci still under treatment [14], maintaining high coverage will be especially important in the endemic area of the Amazon region (Yanomami area) on the border shared by Brazil and Venezuela. The geographically hard to reach location of the affected area coupled with the migratory nature of the population make it especially difficult to maintain regular treatments. In some cases, use of an alternate therapy, such as the antibiotic doxycycline as an end-game approach, may be necessary to ensure full elimination of infection and transmission. The use of doxycycline in onchocerciasis control programs has been demonstrated in the African setting [15].

Other critical issues remain that must be addressed in order to maintain the momentum towards successful onchocerciasis elimination. Populations at-risk must continue to be accurately counted and located, to ensure sustained treatment coverage rates in the remaining areas of transmission of the disease. People living in endemic areas must continue to participate in educational programs in order to understand issues relating to stopping treatment and the surveillance necessary to help identify potential recrudescence. There must be support for post-treatment surveillance and evaluations once treatment has been suspended in order to ensure and confirm transmission elimination. To accomplish this, new or improved diagnostic tools need to be developed and made available. Concerted attention to these issues by all partners, with the critical backing of expanded political will and financial support from governments in affected countries, is necessary to maintain the momentum and assure completion of this public health program [16].

## Adios, onchocerciasis

Certification by the World Health Organization of the elimination of onchocerciasis in Colombia in late 2011 or early 2012 would serve as a test case for both the operational approach taken to reach the point of elimination, and for the method by which elimination is certified. If the initiative proves successful on a regional level, it would represent a true international public health triumph -- the end of nearly half a millennium of onchocerciasis in the Western Hemisphere. More than half a million people would be forever free from the threat of contracting this debilitating and blinding disease. Moreover, the elimination of onchocerciasis carries with it significant social and economic benefits for the formerly affected individuals, communities, and countries.

In a fitting historical counterpoint, the lessons learned through the elimination of onchocerciasis in the Americas can be applied to the African setting. Although the challenges of onchocerciasis elimination are more daunting in Africa due to the size of the affected population and the vast geographic extent of the disease, recent studies suggest the feasibility of elimination in some areas using current treatment tools [17].

## Conclusions

Onchocerciasis has been a public health threat in the Americas for nearly five centuries, impacting hundreds of thousands of people with the threat of severe dermatological conditions, vision impairment and blindness. However, advances in pharmaceutical therapy and innovative approaches to public health developed by various stakeholders have established an effective approach to addressing the disease on individual, community, national and regional levels. As a result of continued and focused investment from a range of contributors, including government, non-profit, scientific and industry bodies, there is very real potential for elimination of onchocerciasis from the Western Hemisphere within the next few years. Such an accomplishment would hold tremendous public health value for the region as well as offer valuable lessons learned for other geographic areas where onchocerciasis continues to be a health burden.

## Competing interests

KG is employed by Merck, which donates MECTIZAN and provides financial support for the MECTIZAN Donation Program. AH is director of the MECTIZAN Donation Program which receives funding from Merck. MS coordinates regional activities through the Onchocerciasis Elimination Program for the Americas (OEPA), employing MECTIZAN donated by Merck, and OEPA through the Carter Center has received some financial support via Merck and the MECTIZAN Donation Program.

## Authors' contributions

KG developed the overall concept of the review, conducted the historical research, proposed the future implications of onchocerciasis elimination beyond the Americas, and drafted the manuscript. AH provided input on the operational elements of global river blindness programs and informed the medical and scientific aspects of ivermectin use and onchocerciasis control and elimination. MS elaborated on the details of the country-specific programs including treatment statistics, mapping, and criteria for disease elimination, and provided insights on onchocerciasis from a Latin America perspective. All authors read and approved the final manuscript
